# Phylogeography of Y-chromosome haplogroup O3a2b2-N6 reveals patrilineal traces of Austronesian populations on the eastern coastal regions of Asia

**DOI:** 10.1371/journal.pone.0175080

**Published:** 2017-04-05

**Authors:** Lan-Hai Wei, Shi Yan, Yik-Ying Teo, Yun-Zhi Huang, Ling-Xiang Wang, Ge Yu, Woei-Yuh Saw, Rick Twee-Hee Ong, Yan Lu, Chao Zhang, Shu-Hua Xu, Li Jin, Hui Li

**Affiliations:** 1 MOE Key Laboratory of Contemporary Anthropology, School of Life Sciences, Fudan University, Shanghai, China; 2 Institut National des Langues et Civilisations Orientales, Paris, France; 3 Saw Swee Hock School of Public Health, National University of Singapore, Singapore, Singapore; 4 Chinese Academy of Sciences and Max Planck Society (CAS-MPG) Partner Institute for Computational Biology, Shanghai Institutes for Biological Sciences, Chinese Academy of Sciences, Shanghai, China; 5 University of Chinese Academy of Sciences, Beijing, China; 6 School of Life Science and Technology, ShanghaiTech University, Shanghai, China; 7 Collaborative Innovation Center of Genetics and Development, Shanghai, China; Estonian Biocentre, ESTONIA

## Abstract

Austronesian diffusion is considered one of the greatest dispersals in human history; it led to the peopling of an extremely vast region, ranging from Madagascar in the Indian Ocean to Easter Island in Remote Oceania. The Y-chromosome haplogroup O3a2b*-P164(xM134), a predominant paternal lineage of Austronesian populations, is found at high frequencies in Polynesian populations. However, the internal phylogeny of this haplogroup remains poorly investigated. In this study, we analyzed -seventeen Y-chromosome sequences of haplogroup O3a2b*-P164(xM134) and generated a revised phylogenetic tree of this lineage based on 310 non-private Y-chromosome polymorphisms. We discovered that all available O3a2b*-P164(xM134) samples belong to the newly defined haplogroup O3a2b2-N6 and samples from Austronesian populations belong to the sublineage O3a2b2a2-F706. Additionally, we genotyped a series of Y-chromosome polymorphisms in a large collection of samples from China. We confirmed that the sublineage O3a2b2a2b-B451 is unique to Austronesian populations. We found that O3a2b2-N6 samples are widely distributed on the eastern coastal regions of Asia, from Korea to Vietnam. Furthermore, we propose- that the O3a2b2a2b-B451 lineage represents a genetic connection between ancestors of Austronesian populations and ancient populations in North China, where foxtail millet was domesticated about 11,000 years ago. The large number of newly defined Y-chromosome polymorphisms and the revised phylogenetic tree of O3a2b2-N6 will be helpful to explore the origin of proto-Austronesians and the early diffusion process of Austronesian populations.

## Introduction

The origin and differentiation of Austronesian populations and their languages has long fascinated linguists, archaeologists, and geneticists. It is generally accepted that Austronesian populations and their languages originated in Taiwan, where the highest diversity in language in the Austronesian language family is observed [1, 2]. The “express train” model of the Austronesian expansion [3], also known as the “Out-of-Taiwan” model, postulates that the earliest archaeological culture in Taiwan, the Dabenkeng culture (about 3000 BC to 2500 BC), was created by ancestors of Austronesian populations. Migrants from Taiwan started a trek that eventually led to the colonization of Island Southeast Asia, Madagascar, Melanesia, and Remote Oceania [4, 5]. The “express train” model is the majority view in the field, however there are alternative hypotheses, like the “entangled bank” model [6] and “slow boat” model [7]. Of note, the most advanced research from a linguistic perspective[8], genome-wide data[9, 10], and ancient DNA[11] tends to support the “express train” model-.

Nevertheless, the origin of the Dabenkeng culture, the earliest Neolithic archaeological culture in Taiwan, is still unclear [12, 13]. The Dabenkeng culture appeared at about 3000 BC and spread quickly around the western coast of the Taiwan island as well as to the Penghu Islands [14]. Even though archaeological dating of the Dabenkeng culture does not support an origin before 3000 BC, some scholars have argued that it might have originated as early as 4000 BC [15]. Remains of rice and millet have been found in archaeology sites of the Dabenkeng culture [15]. Most scholars believe that the migration of the descendants of Dabenkeng populations from Taiwan to the islands of Southeast Asia triggered the great dispersal of Austronesian populations [3, 12]. The Dabenkeng culture may have close connections with Neolithic sites on the coastline of Fujian province, such as the Keqiutou and Fuguodun sites [13]. Some archaeologists have also argued that traits found in Keqiutou, Fuguodun, and sites of the Dabenkeng culture can be traced back to the Hemudu and Majiabang cultures of the lower Yangtze area [13]. However, archaeological evidence has not clarified the direct precursor of the Dabenkeng culture [15].

The appearance of foxtail millet in the Dabenkeng culture also remains mysterious [3, 14]. According to archaeological studies, foxtail millet (*Setaria italica*) was domesticated about 11,000 years ago in northern China [16]. It then became the dominant food crop in the region in the Neolithic age [17]. By contrast, there is no evidence for foxtail millet in South China in the Neolithic age, where rice is the dominant food crop [18]. Of note, millet still plays an important role in traditional activities of modern Taiwan aborigines, such as festivals, ceremonies, and sacrifices to their ancestors [19, 20]. Based on these facts, some linguistic researchers have proposed a link between the Dabenkeng culture and ancient Neolithic cultures in northern China, where millet has been the main source of food for thousands of years [17, 21]. It is proposed that a group of ancient populations with foxtail millet planting techniques left the coastal regions near the Shandong and Jiangsu Provinces in North China and moved southward along the coastline. They may have undergone cultural and gene exchange with ancient populations of the Hemudu culture and Majiabang culture in the Yangtze River delta and Hangzhou Bay. This ancient population may have arrived at both coasts of the Taiwan Strait at about 5,000 years before the present and created the earliest Neolithic sites in the region, like those found in Fujian province and Dabenkeng cultures in Taiwan [17, 21]. However, the general characteristics of Austronesian populations only appeared after the emergence of Neolithic sites on both sides of the Taiwan Strait at about 5,000 years before present. Before this time, ancestral groups of Austronesians should be treated as ordinary Neolithic populations in the eastern part of East Asia, rather than as Austronesian populations.

Genetic studies of uniparental marker (Y-chromosome and mtDNA) have provided a highly complex scenario for the diffusion process of Austronesian populations and their languages [7]. Full mtDNA sequences have been available for decades and have been used to identify specific mtDNA lineages in local populations. Previous studies have shown that the maternal haplogroup B4a1a with the "Polynesian motif" is the predominant lineage in Austronesian populations from Remote Oceania [22]. Trejaut *et al*. indicated that the mtDNA "Polynesian motif" is derived from Taiwan, thus supporting the “Out-of-Taiwan model” [22]. However, a comprehensive study by Soares *et al*. suggested that the common ancestor of Taiwan and Island Southeast Asia populations was established before the Neolithic [23]. Additionally, there are clear signals of two minor Late Holocene migrations via Taiwan. Brandão e*t al*. showed that approximately 20% of mtDNA lineages in the modern Island Southeast Asia gene pool resulted from an "out-of-Taiwan" dispersal, and most of the remaining lineages resulted from earlier processes [24].

The ages of currently-used Y-chromosome markers (e.g., O1-M119, O2a-M95, and O3-M122) are too old to distinguish paternal lineages of Austronesian populations from different regions [25]. Previous studies have shown that O1-M119, O2a-M95, and O3-M122 may be the predominant paternal lineages in the ancestors of Austronesian populations, while C2-M38, K*-M9, S-M230, and M-M4 are consistent with admixture of local populations during the Austronesian expansion process- [26]. A discontinuous distribution of paternal lineages was found in Austronesian populations. In populations from Polynesia and Remote Oceania, O3-M122, C2a-M208, S-M230, and M-M4 are the four most frequent paternal lineages [26, 27].O1a-M119, O2a-M95, O3-M122, and K*-M9 are the predominant lineages in western Austronesians [25, 27]. The haplogroup O3-M122 only represents a minor portion of the paternal gene pool of Taiwan’s aborigines [28]. The general distribution of haplogroup O3-M122 in Austronesian populations suggests a strong founder effect in this lineage during the diffusion of Austronesian populations, especially in Remote Oceania [27].

According to previous -studies, the major components of haplogroup O3-M122 in Austronesian populations -were shown to be O3*-M122+, M324+, P201+, P164+, 002611-, M7-, and M134- [26–35]. In this paper, we named this haplogroup as “O3a2b*-P164(xM134)”. However, no unique Y-chromosome polymorphic markers have been found for O3-M122 in Austronesian populations until 2015 [36]. Haplogroup O3a2b*-P164(xM134) is one of the predominant paternal lineages in the Ami population from Taiwan and most Austronesian populations from Remote Oceania [28, 31]. Hence, we can conclude that haplogroup O3a2b*-P164(xM134) is an important lineage to understand the origin of Austronesian populations. However, few Y-chromosome single nucleotide polymorphisms (Y-SNP) have been determined for this haplogroup and the internal structure of this haplogroup remains unknown. In this study, we provide a series of newly discovered Y-SNP markers and a revised phylogenetic tree, and clarify the phylogeographic distribution of sub-branches within this haplogroup.

## Materials and methods

Blood or saliva samples from populations in East Eurasia were collected from unrelated healthy males over the past 10 years. All individuals were adequately informed and signed their informed consent forms before their participation. The ethics committee for biological research at the School of Life Sciences in Fudan University approved the study (certificate number: FDEL2012-163). Genomic DNA was extracted using the DP-318 Kit (Tiangen Biotechnology, Beijing) according to the manufacturer’s protocol. Seventeen Y-short tandem repeat markers (Y-STR) (DYS19, DYS389I/II, DYS390, DYS391, DYS392, DYS393, DYS437, DYS438, DYS439, DYS448, DYS456, DYS458, DYS635, Y-GATA H4, and DYS385a/b) were amplified using the AmpFlSTR® YFiler™ PCR Amplification Kit (Applied Biosystems, Foster City, CA, USA). Amplification products were analyzed using the ABI 3730 and ABI 3130 Genetic Analyzers (Applied Biosystems). Electrophoresis results were analyzed using Genscan v. 3.7 and Genotyper v. 3.7 (Applied Biosystems). Y-STR data of haplogroup O3a2b*-P164(xM134) and O3a-M324*(xM7,M134) in Austronesian populations were also collected from the literature [28, 30, 31]. According to Mirabal *et al*. [27], O3a-M324*(xM7,M134) samples from Austronesian populations in Oceania should actually be O3a2b*-P164(xM134). Therefore, Y-STR from Delfin *et al*. [30] were also included in this study. The haplotypes for which data was available for all 15 Y-STR polymorphisms (excluding DYS385a/b from 17 Y-STRs) were used to construct the median-joining network using the program NETWORK 5.0.0.0 (Fluxus Engineering) [37]. Furthermore, 1,007 Y-SNP markers that covered the major paternal haplogroups of males worldwide and downstream sublineages of East Asia haplogroups were selected. A series of bait libraries were designed to capture the sequences at the 1,007 Y-SNP positions. The DNA extracted from studied samples was sent for next-generation sequencing on the Illumina HiSeq2000 platform (Illumina, San Diego, CA, USA). We used the procedure that we described previously for the other steps prior to next-generation sequencing, i.e. DNA shearing, adding an adaptor, and gel electrophoresis[38]. Typical procedures were used for next-generation sequencing analyses and the determination of genotypes and haplogroups for each sample [39, 40]. A number of samples from East Asian populations belonged to several sublineages of O3a2b*-P164(xM134). A detailed summary of Y-SNP genotyping results for all O3a2b*-P164(xM134) samples from our lab is provided in S1 Table.

To construct the phylogeny of haplogroup O3a2b*-P164(xM134), seventeen available Y-chromosome sequences, including data from our lab, the SGVP project, and other publications, were included. Two data sets were obtained from Yan *et al*. [38], three from Lippold *et al*. [41], one from Kim *et al*. [42], two from Karmin *et al*. [36], three from 1000 Genomes Project [43], one from Lu *et al*. [44] and five from the SGVP project [45]. As described in our previous work [38], variants were called according to a series of criteria [39, 46], including base coverage (> = 3), base quality (>20), and distance between SNPs (>10 bp).The results of SNP calling are summarized in S2 Table. Of note, only vcf files are available for samples from Lippold *et al*. [41] and Kim *et al*. [42]. Hence, the haplogroup of these samples were determined by comparison to the results of other samples. The regulations proposed by the Y-Chromosome Consortium (YCC) [47] were followed when constructing the phylogenetic trees, including definitions of private/non-private polymorphisms (SNP), nomenclature of newly discovered sublineages, and renaming of old haplogroups. However, the sequenced region of samples varied widely among sources. For example, the sequenced region (0.5 Mbp) for samples from Lippold *et al*. [41] is much smaller than that of other sources. Thus, alleles could not be determined at all polymorphic sites for these samples. These samples were indicated on the revised phylogenetic tree with a note.

## Results

**Fig 1:**
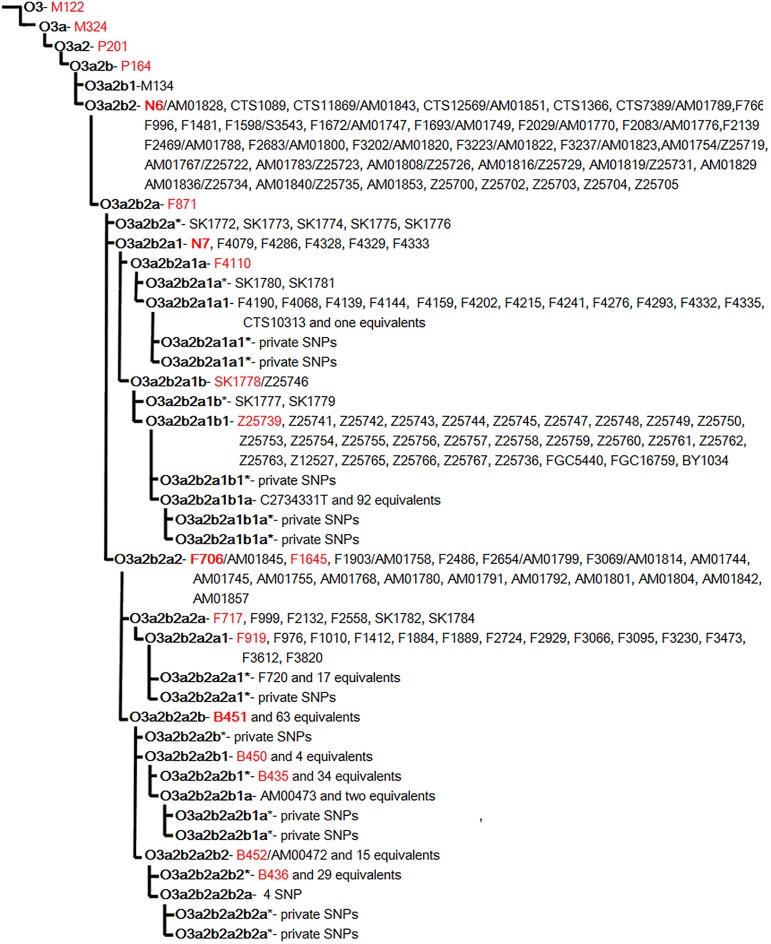
The revised phylogenetic tree of the Y-chromosome haplogroup O3a2b2-N6.

Since all available O3a2b*-P164(xM134) samples had the derived state at marker N6, we redefined this haplogroup as O3a2b2-N6. The revised phylogenetic tree of haplogroup O3a2b2-N6 contained 34 subclades, 310 non-private polymorphisms, and a number of private mutations (Fig 1, S3 Table). The geographic locations of the studied samples and their Y-STR are also included in S3 Table. Detailed information for all Y-chromosome polymorphisms within haplogroup O3a2b2-N6 is provided in S4 Table. All samples from Austronesian populations belonged to sublineage O3a2b2a2-F706, and sublineage O3a2b2a2b-B451 was unique to Austronesian populations. All samples from mainland Asia belonged to other branches of O3a2b2-N6. The most closely related group to Austronesian-specific O3a2b2a2b-B451 was the newly defined subclade O3a2b2a2a-F717.

**Fig 2:**
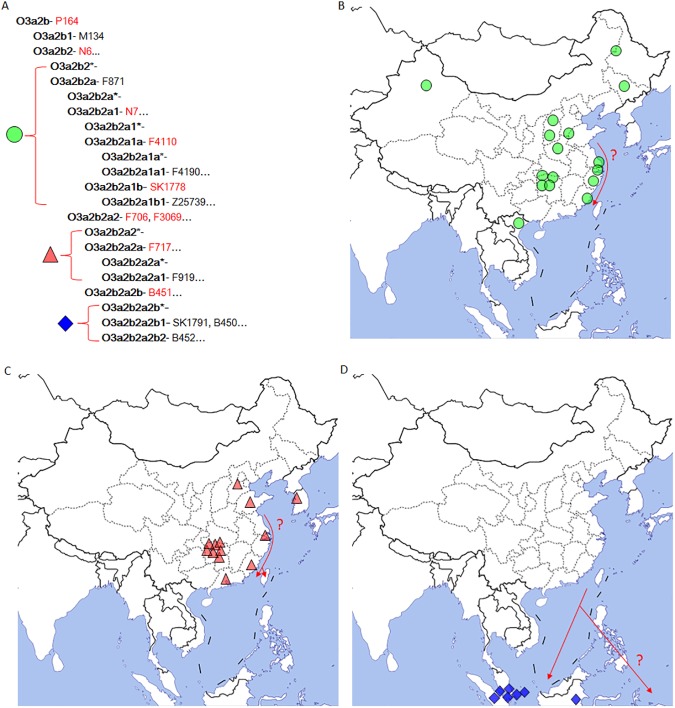
The phylogeographic distributions of sublineages of haplogroup O3a2b2-N6.

The revised phylogenetic tree and genotyping results reveal a clear phylogeographic pattern of paternal haplogroup O3a2b2-N6. We separated all N6+ samples into three categories: O3a2b2*-N6(xF706), O3a2b2a2*-F706(xB451), and O3a2b2a2b-B451 (Fig 2A). Haplogroup O3a2b2-N6(xF706) included samples from either North China or the southeast coastline of East Asia, except for four samples from Hunan province in central China (Fig 2B). Most importantly, we found that O3a2b2a2*-F706(xB451) samples tended to appear on the eastern coastal regions of Asia, except for seven samples from Hunan province in central China (Fig 2C). All samples of O3a2b2a2b-B451 were from Austronesian populations (Fig 2D). Based on the revised tree, we observed that the phylogenetic relationships among O3a2b2-N6 samples from mainland Asia are complex, even though the haplogroup frequencies of O3a2b2-N6 in populations of mainland Asia were low (2.23% in all of our samples, details of N6+ samples listed in S3 Table). All samples from Austronesian populations belonged to haplogroup O3a2b2a2b-B451, which was defined by 64 equivalent polymorphisms. These results indicated that haplogroup O3a2b2a2b-B451 was shaped by a strong founder effect within Austronesian populations after a long-term bottleneck, reflecting either long-term isolation or long-distance migration.

**Fig 3:**
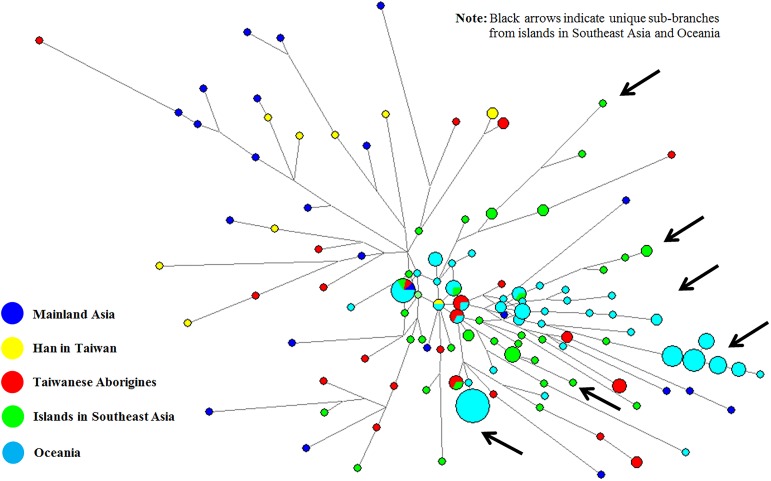
Y-STR network of haplogroup O3a2b*-P164(xM134).

The Y-STR network of haplogroup O3a2b*-P164(xM134) (equivalent to haplogroup O3a2b2-N6 in this study) also provides clues to understanding the internal diversification of this paternal lineage. As shown in Fig 3, samples from the mainland of East Asia and Taiwanese Aborigines are scattered widely on the network. Most of samples from the mainland of East Asia and Han in Taiwan form one cluster while samples from Island Southeast Asia and Oceanians constitute another. This scenario indicates that there was limited contact between populations in the mainland of East Asia and Austronesian populations after their initial splitting. Of note, there are several sub-branches of the Y-STR haplotype that are unique to samples from islands in Southeast Asia and Oceania (Fig 3, indicated by arrow in black). This suggests that there may be several special sub-clades of haplogroup O3a2b2-N6 in Austronesian populations from islands in Southeast Asia and Oceania. However, further genotyping and Y-chromosome sequencing are needed to determine the distribution and the age of these possible sub-clades in Austronesian populations.

## Discussion

In this study, we provided a revised phylogenetic tree of haplogroup O3a2b2-N6 with a large number of newly defined subclades and newly discovered polymorphisms. Genotyping these newly discovered polymorphisms for large samples of Austronesian populations will be useful to determine the distribution of O3a2b2a2b-B451 sublineages within Austronesian populations. As indicated by the Y-STR network (Fig 3), there may be several particular sub-clades of O3a2b2-N6 in in Austronesian populations from Island Southeast Asia and Oceania. Additionally, whole Y-chromosome sequences of O3a2b2-N6 samples will enable accurate estimates of the timing of the origin and internal differentiation of O3a2b2a2b-B451, as well as other possible special sub-clades, in Austronesian populations, similar to previous analyses of haplogroup N-M231 in Uralic populations [48]. Additional genotyping and sequencing results will help clarify the demographic history of Austronesian populations, especially those from the Remote Oceania region.

In the phylogeographic analysis of paternal haplogroup O3a2b2-N6, we observed that haplogroup O3a2b2a2b-B451 is unique to Austronesian populations, while other subclades of O3a2b2-N6 include distant relatives of Austronesian populations in mainland East Asia. According to Karmin *et al*., haplogroup O3a2b2-N6 split from haplogroup O3a2b1-M134 about 20,773 ± 1588 years ago, while the internal differentiation time of O3a2b2a2b-B451 is about 5,511 ± 1068 years ago. The age of O3a2b2a2b-B451 is highly consistent with that proposed by archaeologists and linguists who claim that the Dabenkeng culture (3000 BC or older) was created by direct ancestors of Austronesian populations. Hence, our results support those of Mirabal *et al*., who proposed that haplogroup O3a2b2a2b-B451 is one of the founding paternal lineages of Austronesian populations [27]. Haplogroup O3a2b2a2b-B451 underwent a successful expansion about 5,500 years ago and subsequently became a predominant paternal lineage in Austronesian populations from Taiwan to Remote Oceania.

Furthermore, the geographic distribution of O3a2b2*-N6(xB451) samples provides insight into the origin of haplogroup O3a2b2a2b-B451. Except for eleven males from Hunan, all O3a2b2*-N6(xB451) samples were from North China and the eastern coastal regions of East Asia (Fig 2B and 2C). We found that the Y-STR haplotypes of O3a2b2a2a-F717 males from Hunan are positioned very close to each other (S3 Table). Thus, the overrepresentation of this haplogroup in Hunan could be explained by the possible recent expansion of minor sub-lineages of haplogroup O3a2b2-N6. Since no remains of foxtail millet have been found in archaeology work in Hunan, we consider that the overrepresentation of haplogroup O3a2b2-N6 in populations in Hunan and Austronesian populations may indicate-sharing of a heritage from a common ancestral group, rather than a direct connection between this two populations.

The connection of Austronesian-specific O3a2b2a2b-B451 with modern samples from the coastline of North China could explain the appearance of foxtail millet *(S*. *italica* L. Beauv.*)* in the Dabenkeng culture in Taiwan. Given that rice is the dominant food crop in South China [18], the appearance of complex foxtail millet planting technology in the Dabenkeng culture in Taiwan suggests a migration of populations from North China along the eastern coastline of Asia [17, 21]. Hence, we deduced that the paternal lineage O3a2b2a2b-B451 may be derived from ancient populations in North China, where foxtail millet was the dominant food crop. The appearance of the paternal lineage O3a2b2a2b-B451 in the common ancestors of Austronesian populations may correspond with the introduction of foxtail millet to Neolithic cultures of Taiwan. However, there is another diffusion route of foxtail millet into the southern region of Yangtze River. Foxtail millet diffused southward along the eastern edge region of the Tibetan Plateau at about 5000 years ago [49] and subsequently appeared in Thailand at about 4500–4000 years ago [50]. However, it is generally accepted that the southward diffusion of foxtail millet along the eastern edge region of the Tibetan Plateau is due to South Tibeto-Burman populations [51]. Also, the age of foxtail millet in Thailand is later than those found in Taiwan (5000 years ago or even earlier). Therefore, we can generally rule out the possible migration from Thailand to Taiwan in prehistoric ages.

Due to sea level rise during the Holocene marine transgression in East China, the archaeological traces of the migration of ancestors of Austronesians along the coastline may now be under water [52, 53]. Thus, genetic studies are an effective way to investigate the history of Austronesian ancestors before they appeared in Taiwan. However, linguists have proposed several linguistic connections between ancestors of Austronesian languages and ancestors of Sino-Tibetan languages in North China in the Neolithic age [17, 21]. According to our study, the closest relatives of O3a2b2a2b-B451 were found in North China or locations on the eastern coastal regions of Asia, despite low frequencies of O3a2b2-N6 in populations from these locations. The results of genetic analyses in this study were to some degree consistent with the results of linguistic analyses. However, the deductions in this study are based on Y-chromosome evidence, which is only relevant to the direct paternal line. Studies of maternal mtDNA and whole genomes data should help to confirm our conclusions. More importantly, ancient Y-chromosome DNA, mtDNA, and whole genome sequences of human remains of the Dabenkeng culture may provide the definitive key to unraveling the origin of proto-Austronesians populations.

In conclusion, our genetic studies provide a highly revised phylogenetic tree of haplogroup O3a2b2-N6 and a clear phylogeographic pattern of its sublineages. We determined that sublineage O3a2b2a2b-B451 is unique to Austronesian populations, while close relatives of this sublineage were mainly found in North China or locations on the eastern coastal regions of Asia. We proposed that sublineage O3a2b2a2b-B451 represents the genetic connection between ancestors of Austronesian populations and ancient populations in North China, where the domestication of foxtail millet occurred about 11,000 years ago. Ancient DNA studies and additional genotyping of newly discovered polymorphisms and whole Y-chromosome sequencing of modern O3a2b2-N6 samples will clarify the dispersal and expansion patterns of Austronesian populations in the future.

## Supporting information

S1 Table Summary of Y-SNP genotyping results for O3a2b*-P164(xM134) samples from our lab. (XLSX)

S2 Table VCF file for SNP calling results of sequenced samples. (VCF)

S3 Table Information of samples involved in this study and the updated Y-chromosomal phylogeny of haplogroup O3a2b2-N6. (XLSX)

S4 Table Detailed information of all Y-chromosome polymorphisms within haplogroup O3a2b2-N6. (XLSX)
